# Addition of an Emotionally Stable Node in the SOSa-SPSa Model for Group Emotional Contagion of Panic in Public Health Emergency: Implications for Epidemic Emergency Responses

**DOI:** 10.3390/ijerph17145044

**Published:** 2020-07-14

**Authors:** Xiaoyang Ni, Haojie Zhou, Weiming Chen

**Affiliations:** Faculty of Engineering, China University of Geosciences, Wuhan 430074, China; xy_ni@163.com (X.N.); chenpool@126.com (H.Z.)

**Keywords:** group emotional contagion, numerical simulation, SIR model, public health emergency

## Abstract

Sentiment contagion is similar to an infectious disease that spreads in a crowd. In this study, we explore the law of emotional infection under sudden public events by SIR model. The paper adds an emotionally stable node and establishes a group emotional infection model of U-SOSPa-SPSOa model. Simulation results show that our model is reasonable and can better explain the entire contagion process by considering four groups (unsusceptible-susceptible-optimistic-pessimistic) of people. Our theoretical results show: When the pessimists were below the critical value of 0.34, the number of negative emotional groups first increased and then decreased. As the proportion increases, the emotional peak of pessimists increases. The cure probability *θ_o_* has the least influence on the *P*(*t*), and at the same time, under the action of *θ_p_*, the *P*(*t*) reaches the stable state first. The increase of the risk coefficient can promote the pessimist infection. When the degree of risk is low, the rate of emotional infection is increased. When the degree of risk is high, the rate of infection is slowed. Therefore, system customizers and related managers can improve the efficiency of stable groups, adjust the proportion of initial negative emotions, control the infection of the spontaneous infection process, and directly deal with negative emotions. They can carry out treatment and other means to stabilize group emotions and maintain social stability.

## 1. Introduction

In recent years, there have been many public health emergencies including COVID-19, SARS, H1N1, etc. In addition to the damage caused by the infection itself, group movements in public emergencies often have group emotional and behavioral responses. Emotion is a kind of psychological activity produced by individuals along with cognition and consciousness, which not only affects an individual’s behavior, but also can affect the behaviors of other individuals through emotion contagion. Public health emergency has the characteristics of unpredictability, destructiveness, and uncertainty of evolution. Therefore, it is easier to receive the widespread attention of social groups. The rapid development of the Internet has provided a wide range of channels for the dissemination of information and emotions, making individual fears turn into collective panics, such as the panic buying of toilet paper during the COVID-19 epidemic. In the event of an emergency, it is often difficult for people to have enough time to make a reasonable decision. The behavioral characteristics of individuals tend to be more instinctual and emotional. The group emotions generated by emotional resonance greatly influence the group behavior and its characteristics. The generation and development of group emotions are similar to other communication theories, and they creep into the mutual infection and interaction of individuals in the group. At present, the research on the rules of emotional infection focuses on the emotional infection model combining emotional valence in the group. For example, Faroqi et al. (2015) describe the emotional level in emergencies as: Calm, anxiety, fear, horror, panic, and hysteria [1]. Totterdell and Peter (2000) found that it is easier to achieve consistency with the infected person when the individual was active in the case of emotional infection [2]. The mechanism of emotional consistency is reflected in the ability to imitate, and the rendering of emotion through mutual stimulation and mutual infection based on imitation promotes the generation of consistent emotions. Mimicry represents a family of synchronous behaviors that primarily include facial expressions, but also include body postures, eye movements, speech gestures, and laughter [3,4,5,6]. The above literature analyzes the factors affecting the emotional infection of individuals and considers the degree of influence of different emotional valences on individuals in the crowd. Regarding the identification and simulation of emotional infections, many scholars have combined psychology models and communication models to simulate the process of emotional infection between groups. Rincon et al. establishes individual characteristics with PAD and simulates the emotional infection of heterogeneous groups [7,8]. Tsai et al. proposed the ESCAPE model (evacuation simulation with children, authorities, parents, emotions, and social comparison) on the basis of the ASCRIBE model (describes a contagion model resembling dynamics properties as in thermodynamic systems), taking into account the familiarity and identity of the agent with the environment (family relations, authorities, etc.), and simulated the emotional infection process during airport evacuation [9,10]. The simulation model research is part of the research on emotional infection. Liu et al. (2019) proposed the emotional infection simulation model, considering the importance of the administrator for emotional control, and established a corresponding simulation demonstration prototype system [11]. Li et al. (2013) adopted the agent’s simulation method studies the evolution process of group emotions [12]. Manzoor and Treur (2013) address an agent-based computational social agent model, the agent-based model integrates emotion-related valuing, in order to analyze the role of emotions in socially affected decision making [13]. The model for emotion regulation is based on recent neurological literature which addresses how emotion regulation takes place by an interaction between prefrontal cortex and amygdala [14], social movements driven by emotions [15,16,17], and that emotion contagion plays a crucial role in the spread of these emotions [18]. Some scholars have carried out research on the combination of the transmission mechanism and emotional infection. Bosse et al. proposed a model of emotional absorption based on the thermal conduction mechanism, which applies the law of thermal energy propagation to the process of emotional infection [19,20]. Durupinr found that the emotional characteristics of the group and the virus from the node to the node are very similar in emergency and combined with the infectious disease model to study emotional infection [21,22]. Lungu (2013) established Newton’s emotional model [23] and combining Plutchiks’ theory [24] with Newton’s law. Fu (2014) simulated the dynamic effects of emotional infection and proposed an epidemiology-based model (susceptible infected recovered susceptible, SIRS), they believed that the initial infection rate had little effect on the final stable system, and the group density and individual movement speed could accelerate emotion infection [25]. Mao (2018) regards the emotions in the population as a process of disease transmission to a certain extent, improves on the basis of the SIRS model, and obtains the CA-SIRS model (cellular automaton SIRS). It is divided into three types: Susceptible to infection, infection, and immunity [26]. Wang et al. (2016) enriched the description of crowd emotional infection, joined latent and sensitive groups, and established a sense of vulnerability—latent—infection-recovery-sensitive model (SLIRS) [27]. Cao et al. (2017) established a P-SIS model to further simulate crowd evacuation combined with the Big Five Personality Theory (OCEAN) and the infectious disease SIS model [28]. Most of the studies model the definition of emotional infection mechanism rules from the macro and micro perspectives, and there is a lack of comprehensive and unified understanding. The rules of emotional infection between the crowd and individuals under the refinement of emotional valence need to be further studied to improve the effectiveness of the fusion model.

## 2. Model Basis

Alison et al. (2010), based on data of the Framingham Heart Research Center, showed that people of positive and negative change across social networks as infectious diseases spread in a long period of time [29]. Because the pessimists are very infectious, the mental emotions are very few, and the infection is very weak, and the group emotions tend to be consistent in a short period of time. In the SOSPa-SPSOa model (susceptible-optimistic-susceptible (SOS) and susceptible-pessimistic-susceptible (SPS)), the group was divided into optimists, pessimists, and emotionally susceptible group [30]. The defined emotional infection mechanism is a complex network formed by the relationship between people and people. The three nodes in the network represent three kinds of emotional states that may exist. There are several types of state contacts in the network.

(1) SO (infects between susceptibility and optimists). When the optimist comes in contact with the susceptibility, the susceptibility becomes optimistic with a possibility of *β_o_*. Regardless of whom the susceptibility comes in contact with, the susceptibility spontaneously becomes optimistic at a rate of *α_o_*. SP (susceptibility infects with pessimists). Regarding the susceptibility confronting the pessimists, while the susceptibility reverts to being pessimistic at a rate of *β_p_*, the susceptibility is spontaneously infected by the pessimists at a rate of *α_p_* regardless of contacts.

(2) PO (infects between pessimists and optimists. When the pessimists come in contact with the optimists, the possibility of the pessimists turning into optimists is *m*_2_, PS (infects between optimists and susceptibility). When the pessimists come in contact with the optimists, the possibility of the pessimists will turn to the susceptibility is *m*_1_, and regardless of whom the pessimists come in contact with, the possibility of *r_p_* that the pessimists become susceptibility exists.

(3) OP (infects between optimists and pessimists). When the optimists come in contact with the pessimists, the possibility of the optimists turning into pessimists is *l*_2_. OS (infects between optimists and susceptibility). When the optimists come in contact with the pessimists, the possibility of the optimists turning into the susceptibility is *l*_1_, and regardless of whom the optimists come in contact with, the possibility of *r_o_* that the optimists become susceptibility exists.

**Figure 1:**
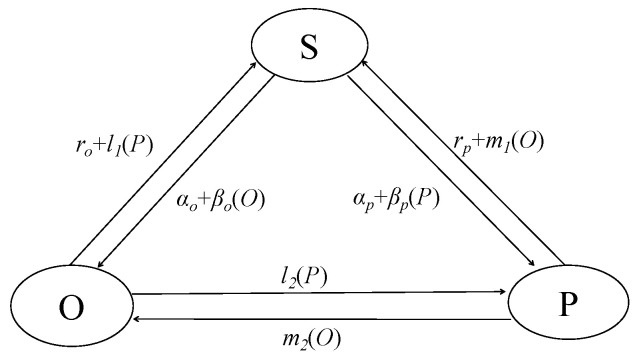
Emotional infection model of SOSPa-SPSOa (susceptible-optimistic-susceptible (SOS) and susceptible-pessimistic-susceptible (SPS)) model.

The individual in the network infects surrounding individuals through contact, the state of each individual conversion is affected by the state of the surrounding nodes in Figure 1 (considering that the SIR model uses *r* to represent the recovery rate, the author uses *g* as the recovery rate to rewrite *r* as the recovery rate).

**Figure 2:**
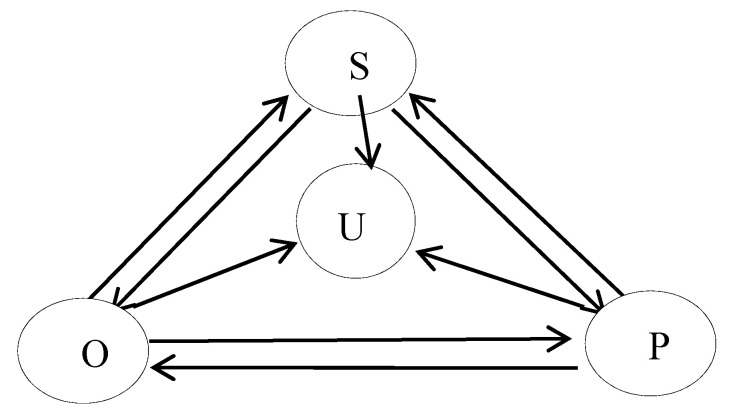
Emotional infection model of U-SOSPa-SPSOa (unsusceptible-susceptible-optimistic-susceptible (SOS) and susceptible-pessimistic-susceptible (SPS)) model.

However, considering the public domain, there are often perfect emergency management systems and preventive measures. Professional security personnel and daily maintenance personnel have corresponding education and training. Therefore, this paper increases the emotional stability group in the emergency and divides the group into emotional stability group and emotion conversion group. The emotionally stable group (*U*) is calm during emergencies, less susceptible to event-related emotions, and plays a role in stabilizing and regulating group emotions. The three state contacts of PU (contact between pessimists and stable emotions), OU (contact between optimists and stable emotions), and SU (contact between susceptibility and stable emotions) are shown in Figure 2.

Secondly, it is considered that the infectious disease model lacks the contact relationship of emotional groups in the process of contact infection, and the individual and the individual within the group need to communicate with facial expressions or speech within a certain distance. The emotional infection is more within the contact of the crowd. Emotional infections occur through communication expressions. Therefore, this paper introduces a contact relationship.

According to Totterdell and Peter (2000), when people are infected with positive emotions, they are more likely to reach a consistent mood with infected people [2]. Individuals often do not have a positive effect on positive emotions in emergencies. A high level of contact rate is conducive to the transmission of positive and stable emotions. The positive and stable emotions in the process of communication often have traditional linear contact with the group. The same emotionally stable group often presents a traditional linear contact relationship in the process of communication. However, when factors of pessimists predominate, individuals have intervention and rejection of factors of pessimists under the mechanism of self-protection and isolation of pessimists. Factors of pessimists in high infection level will slow down the transmission of pessimists, affect the occurrence of infection, and pessimists. The contact relationship between emotion and the group in the process of communication often presents a nonlinear contact relationship. Based on the above assumptions and propagation rules, the existing Dongmei et al. (2007) nonlinear contact rate infectious disease model was established to establish a new emotional infection model [31]. The linear contact relationship in the model with the positive emotional group is:

(1) $$g_{1}(O)S=kOS$$

The linear contact relationship in the model with the emotionally stable population is:

(2) $$g_{1}(U)S=kUS$$

The nonlinear contact relationship with the negative emotion group is:

(3) $$g_{2}(P)S=\frac{kPS}{1+aP^{2}}$$

We define *g*_1_ () is the expression for the linear contact relationship. In the linear relationship, we use the coefficient *k* to represent the contact relationship between the two subjects. We define *g*_2_ () is the expression for the nonlinear contact relationship. In the nonlinear relationship, we use *a*: to risk factor of an emergency, which is determined by the extent to which an emergency affects people. It can be seen from Equation (3) that its equation becomes linear when *a* = 0, indicating that the emotional transmission between the populations has a linear relationship in the non-hazardous situation. When *a* is large enough, the contact relationship is nonlinear. The emotional state of the person is unstable, the individual is eager to seek a safe environment in the case of high risk. The degree of association between the groups is small, which is more in line with the individual’s performance under the strong desire for survival.

## 3. Model and Simulation

**Figure 3:**
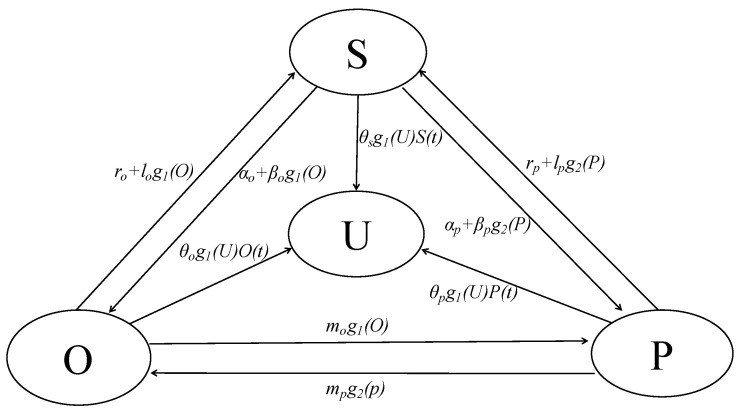
U-SOSPa-SPSOa model with contact relationship infectious disease model.

Based on the infection relationship of the SIR model and SOSPa-SPSOa model, we introduced the contact relationships (1), (2), and (3) of different emotional valencies, and we redefine *l*_1_, *l*_2_, *m*_1_, *m*_2_. Define the infection between optimists and optimists as *m_o_*, *m_p_*, convert optimists and pessimists after infection into susceptibility as *l_o_*, and pessimists and optimists after infection conversion into susceptibility is defined as *l_p_*. A group emotional infection model (U-SOSPa-SPSOa) is established, as shown in Figure 3.

According to the emotional infection model of Figure 3, we add the parameters *θ_s_*, *θ_o_*, *θ_p_* to explore the influence of the interaction between the emotionally stable group (*U*) and the susceptibility (*S*), optimists (*O*), pessimists (*P*) on the mechanism of sentiment contagion and to distinguish which status the infected is prone to transfer when contacting with the opposite status. Combining these numbers, we obtain the equation *P* + *O* + *S* = *N*. We also assume that the two processes of transition from the susceptible subjects to the pessimistic subjects and the optimistic subjects are independent of each other. We know that professionals, such as doctors and police officers, are relatively stable in public health incidents, and they belong to the immunized group within the group. We assume that *U*(*t*) will not revert back to active or susceptible groups. The differential equations of the modified model are as follows:

(4) $$\left\{ \begin{array}{l} \frac{dS(t)}{dt}=r_{o}O(t)+r_{p}P(t)-(\alpha_{o}+\alpha_{p})S(t)+g_{1}(O)\left(l_{0}P(t)-\beta_{O}S(t)\right)+g_{2}(P)\left(l_{p}O(t)-\beta_{p}S(t)\right)-\theta_{S}g_{1}(U)S(t)\\ \frac{dO(t)}{dt}=\alpha_{o}S(t)-r_{o}O(t)-\left(m_{o}+l_{o}\right)g_{1}(O)P(t)+\beta_{o}g_{1}(O)S(t)+m_{p}g_{2}(P)O(t)-\theta_{O}g_{1}(U)O(t)\\ \frac{dP(t)}{dt}=\alpha_{p}S(t)-r_{p}P(t)-\left(m_{p}+l_{p}\right)g_{2}(P)O(t)+\beta_{p}g_{2}(P)S(t)+m_{o}g_{1}(O)P(t)-\theta_{P}g_{1}(U)P(t)\\ \frac{dU(t)}{dt}=\theta_{S}g_{1}(U)S(t)+\theta_{O}g_{1}(U)O(t)+\theta_{P}g_{1}(U)P(t) \end{array}\right.$$

*α_o_*, *α_p_* are the infection probabilities of susceptibility (*S*(*t*)) spontaneously converted to optimists (*O*(*t*)) and pessimists (*P*(*t*)). *β_o_*, *β_p_* are infection probabilities after susceptible groups are exposed to optimists and pessimists. *r_o_*, *r_p_* are optimists and pessimists are restored. *l_o_*, *l_p_* is the probability of infection after conversion between optimists and pessimists, pessimists and optimists into susceptible populations. *m_o_* is the probability of infection after conversion between optimists and pessimists into pessimists. *m_p_* is the probability of infection that is converted into optimists after contact between pessimists and optimists. *θ_o_* is the cure probability of optimists and emotionally stable groups. *θ_p_* is the cure probability of pessimists and emotionally stable groups. *θ_s_* is susceptible the probability of cure for emotional groups and emotionally stable groups. *t* is time.

The model simulation process parameter settings refer to the research data of the Framingham Heart Research Center [29], some parameters of the model are set as follows: *α_o_* = 0.18, *β_o_* = 0.02, *r_o_* = 0.088, *l_o_* = 0.13, *m_o_* = 0.09, *α_p_* = 0.04, *β_p_* = 0.04, *r_p_* = 0.13, *l_p_* = 0.07, *m_p_* = 0.009, and in addition, we set *θ_s_* = 0.02, *θ_o_* = 0.01, *θ_p_* = 0.005. In the emotional infection model, due to the hazard of emergencies, it is assumed that the initial time in the system is negative population *P* = 0.15, emotionally susceptible group *S* = 0.7, positive population *O* = 0.1, *U* = 0.05.

**Figure 4:**
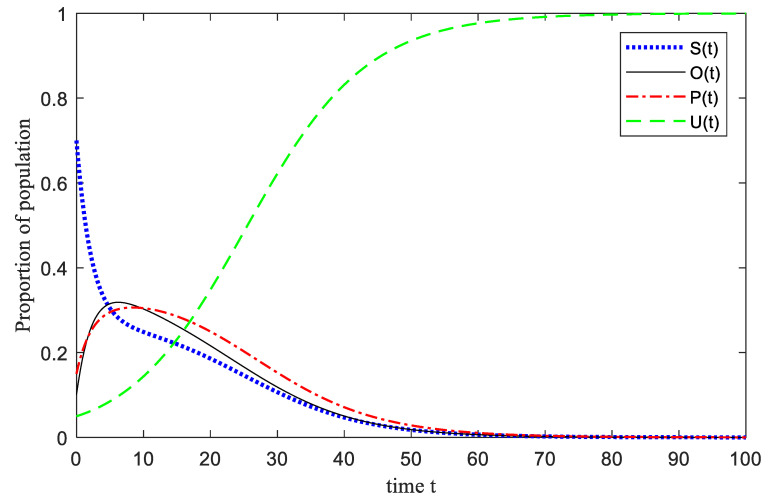
The process of U-SOSPa-SPSOa model.

It can be seen from Figure 4 that the number of *S*(*t*) has a process of a sudden decrease in the short time after the occurrence of an emergency, and the rate of change tends to zero after *t* = 70, and gradually becomes stable. This is because of the influence of the surrounding crowds in the occurrence of an emergency, *S*(*t*) is transformed from an emotionally suppressed state to an emotionally active state, which is transformed into three emotional states. *P*(*t*) rapidly increased from 15% of the initial population to 30% and peaked in a very short time, and then showed a trend of slow decline, and finally reached a steady state. It is because the group is caught off guard in a short period of time, and there are *P*(*t*) such as tension, fear, and pain, and these emotions will produce a “magnification effect” of emotions in the process of spreading and infecting the group, and then Individuals of *P*(*t*) grow rapidly and reach a peak. With the role of *U*(*t*), the proportion of *P*(*t*) begins to gradually decline. For the trend of *O*(*t*) in the picture, it can be seen that the trend is relatively flat in the whole process, even if it has a small range of growth in the initial stage, but the increase is not intense, and then shows a downward trend. Because in the short-term existence of *U*(*t*), *S*(*t*) quickly turn into *O*(*t*), while *O*(*t*) can be quickly converted into *U*(*t*), the trend of *O*(*t*) shows a small increase and then decline. In addition, because of the low initial proportion of *U*(*t*), relative to the initial stage, emotional conversion groups are stimulated to switch quickly under instinct, and the role of stable groups is weak, but with the intervention of *U*(*t*), the group emotions are gradually stabilized. The number of stable groups has increased significantly, finally, the group emotions have returned to stability under the gradual control of the situation.

## 4. Discussion of Results

### 4.1. Analysis of the Influence of U(t)

**Figure 5:**
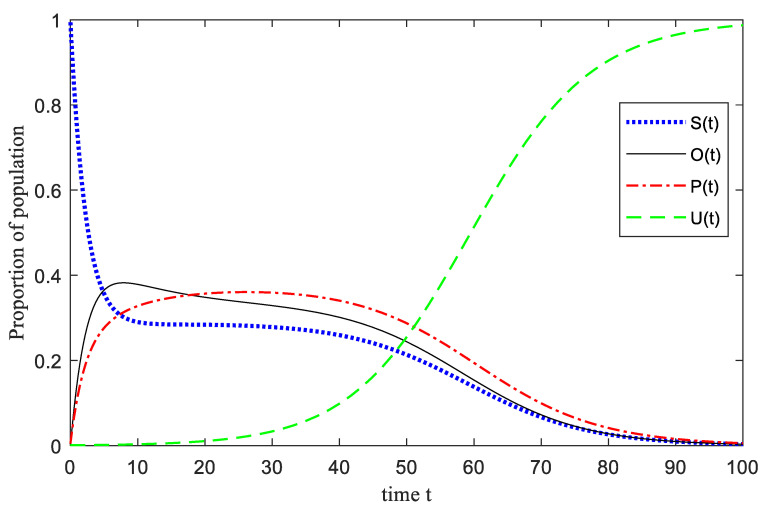
The process of U-SOSPa-SPSOa Model simulation comparison.

**Figure 6:**
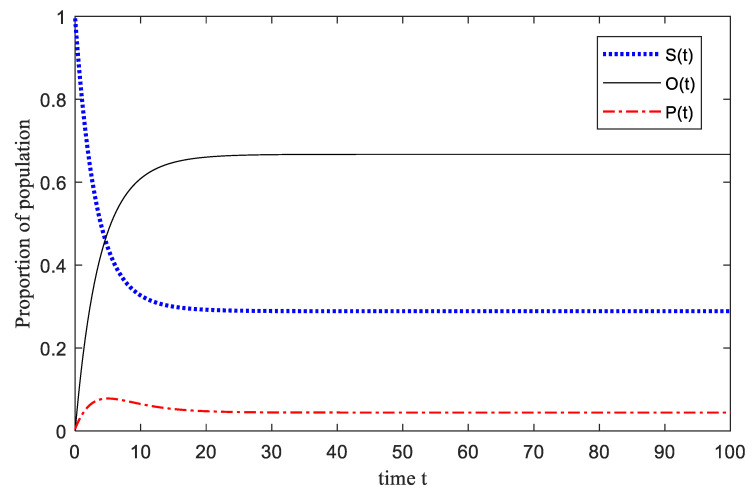
The process of SOSPa-SPSOa Model simulation comparison [31].

The U-SOSPa-SPSOa model considers that the group has a stable group of professional management and maintenance and rescue personnel. We analyze the results of the two states of the emotionally stable group with and without this. Comparing Figure 5 with Figure 6, we can see that the overall change trend of the two figures is similar, and the situation of emotional transformation is similar. Emotionally susceptible groups are quickly converted to other emotional groups in emergencies. However, the overall time of the U-SOSPa-SPSOa model to reach a stable trend is longer than that of the SOSPa-SPSOa model [31], and the proportion of reaching a stable state is also different between the two. (1) The main reason is that the final state of emotional stability set in this article is the immune state. Joining the management and maintenance of the stable group requires more time in the management and maintenance process, especially the professional maintenance and rescue personnel have a certain delay in a more dangerous situation and have a limited role in the initial state. It takes a short time to achieve a rapid and stable role. (2) The proportion of the final stable state is different. The main reason is that without joining the emotionally stable group, the internal optimists play a larger role, but the negative state of the optimists is not fully considered, so the structural response is faster. In the case of joining emotionally stable groups, balanced optimists are converted, and emotionally stable emotions play a key healing role, and the role is higher than optimists, which is more in line with the actual situation. The introduction of emotionally stable groups can make the simulation of group emotions more reasonable under different situations.

### 4.2. Analysis of the Influence of Initially Negative Emotion Scale

**Figure 7:**
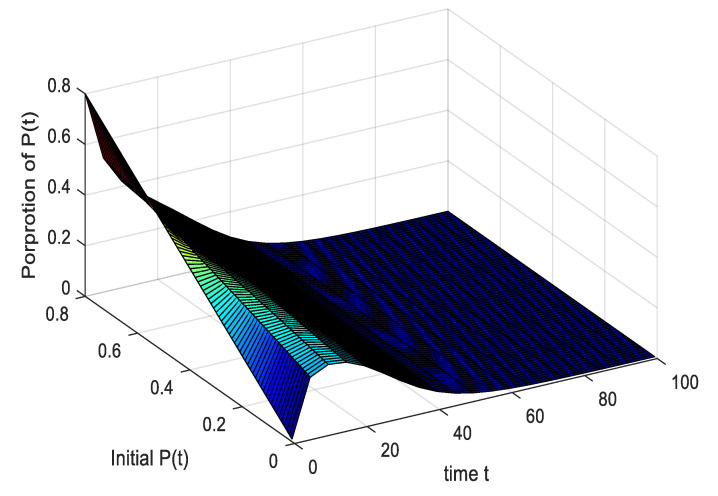
The impact of initial population *P*(*t*) on negative emotional groups.

**Figure 8:**
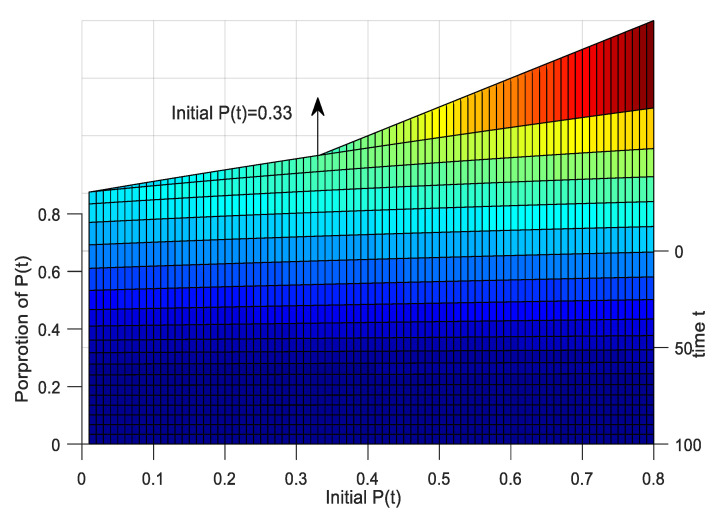
The impact of initial population *P*(*t*) on the pessimists in the main view.

It can be seen from Figure 7 and Figure 8: (1) When the proportion of initially negative emotions gradually increases from 0.01 to 0.8, the time required to reach equilibrium increases significantly, indicating that the *P*(*t*) have a wider range of influences, and the degree of influence is deeper. (2) When the proportion of *P*(*t*) changes from 0 to 0.34, the *P*(*t*) first increase and then decrease, and the emotional peaks of *P*(*t*) increase with the proportion increases, indicating that the increase promotes the spread of negative emotions within the group when the proportion of initial *P*(*t*) is smaller. When the proportion of initially negative emotions increases significantly to above 0.34, the *P*(*t*) dominate in the time, and the rejection of the people within the group causes *P*(*t*) within the group to decline. The results are consistent with the hypothesis and verify the rationality of the model. (3) It can be concluded from this that initial negative emotions below 0.34 belong to medium and low risks, and *P*(*t*) increase steadily, while above 0.34 belong to high-risk states. In this state, the speed of external rescue should be accelerated to fully regulate *P*(*t*) infections in time.

### 4.3. Analysis of the Impact of Spontaneous Infection Process

**Figure 9:**
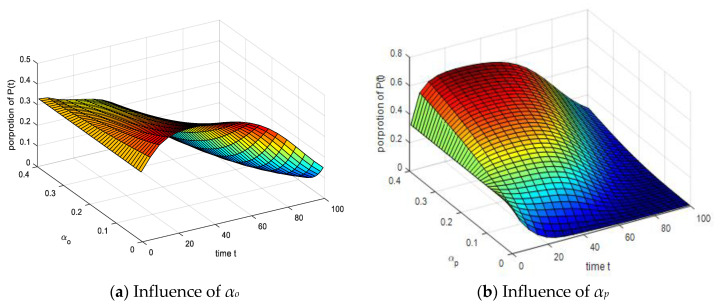
Influence of spontaneous infection probability *α_o_* and *α_p_* on *P*(*t*).

In order to analyze the influence of model parameters on the emotional infection mechanism, and to help identify the results of the population, this paper changes the proportion of the population in the initial emotional state of the population, *S*(*t*) = 0.33, *O*(*t*) = 0.33, *P*(*t*) = 0.33, *U*(*t*) = 0.01. Figure 9a illustrates that the number of parameters declines significantly with the increasing value of *α_o_*. When *α_o_* is small, *P*(*t*) increases first and then decreases, indicating that the overall environment is more pessimistic, and the group’s emotions become more negative. However, as the emotionally stable group comes into play and the group’s confidence is restored, *P*(*t*) will gradually decrease, so it will increase first and then decrease. As shown in Figure 9b, the proportion of *P*(*t*) increases significantly when *α_p_* gradually increases. The main reason is that the increase of *α_p_* gradually increases the probability that *S*(*t*) is converted into *P*(*t*), and the proportion of *P*(*t*) gradually increases. It can be seen from the comparison of the two figures that the effects of *α_o_* and *α_p_* are opposite, and the overall trend shows that the probability of spontaneous infections *α_o_* and *α_p_* have a significant effect on negative groups.

**Figure 10:**
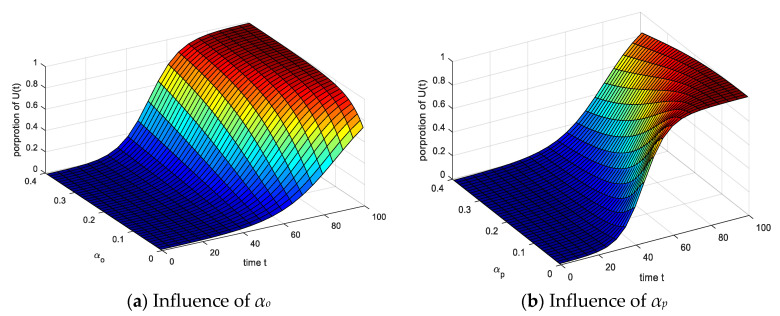
Influence of spontaneous infection probability *α_o_* and *α_p_* on *U*(*t*).

The results of analyzing the influence of spontaneous infection probability *α_o_* and *α_p_* on emotionally stable groups are shown in Figure 10. (1) Under the action of *α_o_* and *α_p_*, the proportion of *U*(*t*) increased significantly over time, indicating that the spontaneity within the group increases with *α_o_* and *α_p_* increase, the group is susceptible to environmental factors, and the exchanges between each other increase, so emotionally stable groups are more likely to play a role. (2) Comparing the effects of *α_o_* and *α_p_* on *U*(*t*), with the increase of *α_o_*, the time of *U*(*t*) decreases when reaching equilibrium, while *α_p_* is the opposite. The increase of *α_o_* indicates that the group’s emotions are more stable, and the S(*t*) are converted into *O*(*t*) and *U*(*t*), the time to reach equilibrium decreases, while the increase of *α_p_* indicates a high spontaneous infection of *P*(*t*) (more dangerous situations). In some cases, group infections will increase significantly, and the results are more in line with the reality that emotional groups in high spontaneous infections are difficult to regulate and *P*(*t*) are difficult to recover in dangerous environments.

### 4.4. Analysis of the Impact of Contact Probability

**Figure 11:**
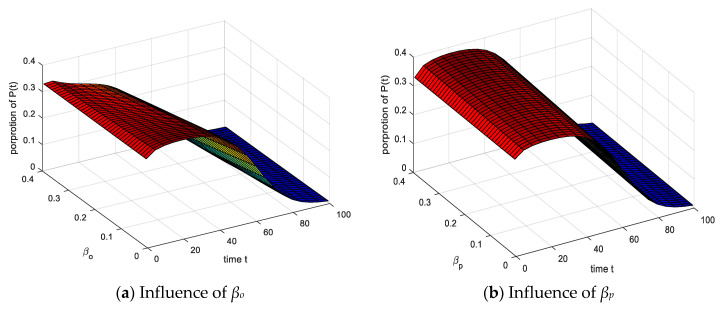
Influence of spontaneous infection probability *β_o_* and *β_p_* on *P*(*t*).

The setting parameters are the same as above. Increasing the probability of contact *β_o_* and *β_p_*, the proportion of *P*(*t*) decreased with the increase of time, the proportion of *U*(*t*) increased, and the time required to reach balance increased in Figure 11. (1) It shows that the emotional infection of the group is strengthened when the contact rate is higher, the more frequent emotional infection prolongs the time of emotional infection. (2) It shows that the contact between groups can alleviate the growth of *P*(*t*), the *O*(*t*) is more effective, and the time required for *P*(*t*) infection increases. The change of equilibrium state is smaller under the comprehensive factors. Compared with Section 4.3 (spontaneous infection process) and Section 4.5 (recovery process), the change range of the result graph is much lower than the spontaneous infection process (Section 4.3) and recovery process (Section 4.5), the impact of the contact process on the group’s emotions is less than the spontaneous infection and recovery process.

**Figure 12:**
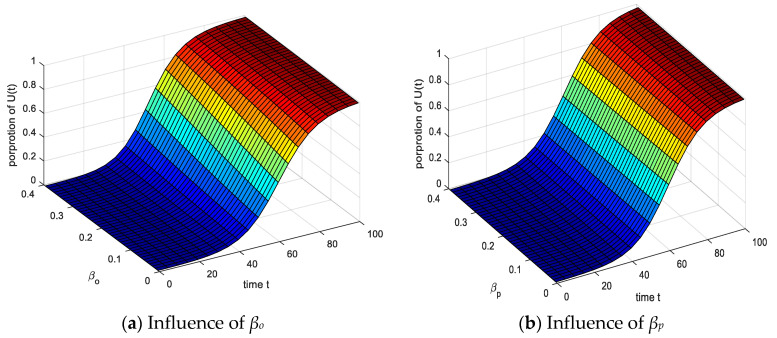
Influence of spontaneous infection probability *β_o_* and *β_p_* on *U*(*t*).

The results of analyzing the influence of the contact probabilities *β_o_* and *β_p_* on U(*t*) are shown in Figure 12. Under the action of *β_o_* and *β_p_*, the proportion of *U*(*t*) increased significantly with time. Comparing the effects of *β_o_* and *β_p_* on *U*(*t*), it can be seen that with the increase of *β_o_*, the time for *U*(*t*) to reach equilibrium gradually decreases, indicating that the probability of positive emotional contact *β_o_* can stabilize the group’s emotions, promoting increase the infection rate of *U*(*t*).

### 4.5. Analysis of the Impact of Recovery Probability

**Figure 13:**
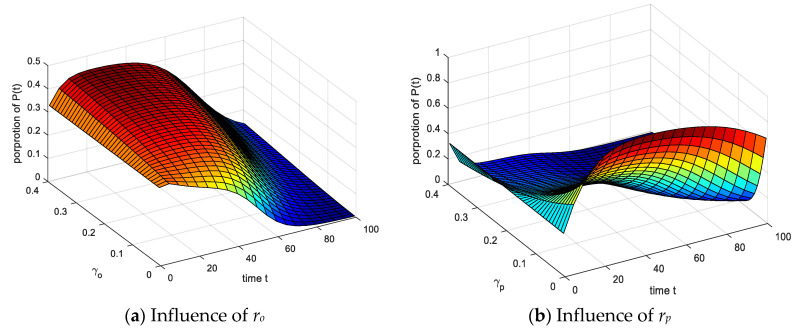
Influence of spontaneous infection probability *r_o_* and *r_p_* on *P*(*t*).

The setting parameters are the same as above. As shown in Figure 13: (1) The proportion of *P*(*t*) decreases significantly and the time to reach equilibrium decreases when the *r_p_* gradually increases, the duration of the entire infection process group decreases significantly, but *r_o_* is opposite, the two inhibit each other. (2) The proportion of the group reaching equilibrium proportion fluctuated significantly, and the *P*(*t*) decreased significantly after a short rise, the increase in the *U*(*t*) was small. It indicates that the group had a good understanding of the outside world and was stimulated by emergencies when the recovery probability *r_o_* and *r_p_* increased.

**Figure 14:**
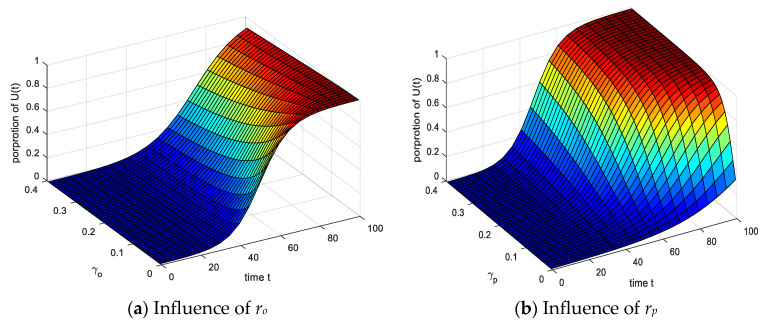
Influence of spontaneous infection probability *r_o_* and *r_p_* on *U*(*t*).

The results of analyzing the impact of infection recovery probabilities *r_o_* and *r_p_* on the *U*(*t*) are shown in Figure 14. (1) Under the action of *r_o_* and *r_p_*, the proportion of *U*(*t*) increased significantly with time. (2) Comparing the effects of *r_o_* and *r_p_* on the *U*(*t*), it can be seen that the time gradually increases with *r_o_* increasing when the model reaches equilibrium. As *r_p_* increases, the time decreases when the model reaches equilibrium. The increase of *r_p_* indicates that the *P*(*t*) have enhanced self-recovery ability, which is converted into *O*(*t*) and *U*(*t*), the balance time is reduced, while *r_o_* is the opposite, *O*(*t*) return to S(t) or other groups. The infection time of the *U*(*t*) increases with the risk of group increased.

### 4.6. Analysis of the Impact of Cure Probability

**Figure 15:**
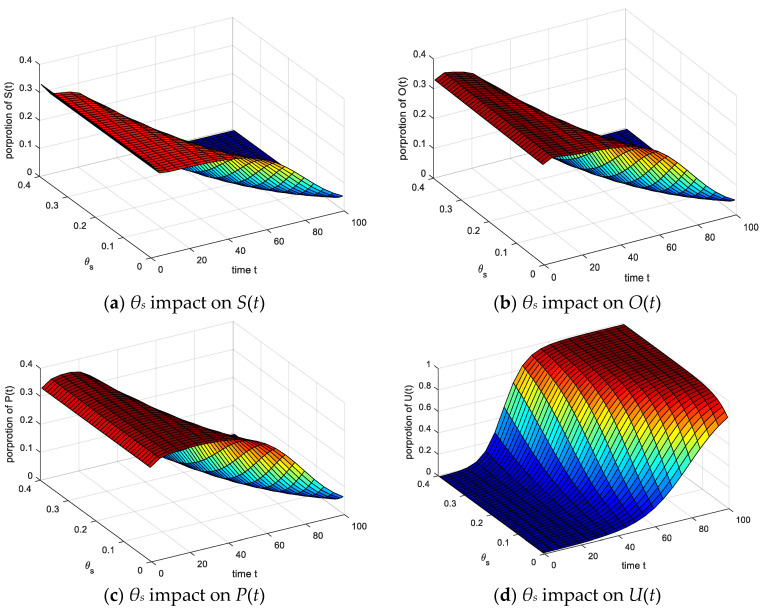
*θ_s_* impact on four emotional groups.

The setting parameters are the same as above. As shown in Figure 15: (1) Comparing the following four figures, it can be found that as the value of *θ_s_* increases, the number of *S*(*t*), *O*(*t*), decreases, *P*(*t*) increases first and then decreases, the *U*(*t*) increases, and *θ_s_* increases exponentially, the speed of each group increases, the time to reach steady state decreases. (2) With the increase of the cure probability *θ_s_*, the effect of the *U*(*t*) is significant, and the direct infection of *S*(*t*) is cured to *U*(*t*), the *S*(*t*), *O*(*t*) and *S*(*t*) are significantly reduced. The response instinct of *S*(*t*) to emergencies makes them turn into *P*(*t*) when the cure probability is low. Therefore, *P*(*t*) increase first and then decrease with the role of stable groups. (3) It is indicated that *θ_s_* promotes group emotional infection and accelerates emotional communication among different groups. However, it can be seen from the change of *P*(*t*) peak that the change of *θ_s_* has little effect on the peak of *P*(*t*).

**Figure 16:**
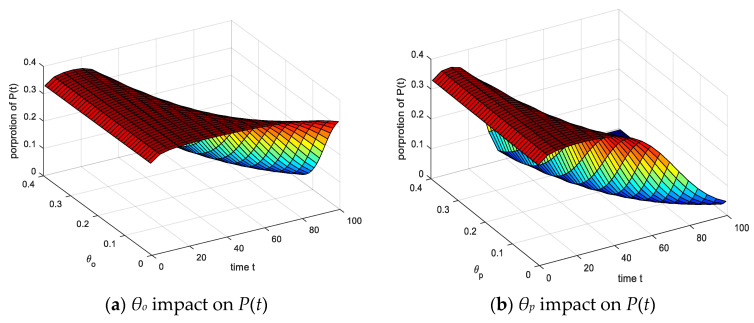
*θ_o_*, *θ_p_* impact on four emotional groups.

It can be seen from Figure 16 that the changes of the variables *θ_o_*, *θ_p_* have little influence on the peak of the *P*(*t*), and the three curves with different values are close to coinciding at the peak. By comparing the influence diagram of *θ_s_*, it can be seen that *θ_o_* has the least influence on the *P*(*t*), and the change range is small. At the same time, under the action of *θ_p_*, the *P*(*t*) reaches the stable state first. It is indicated that the *θ_o_* parameter has a small effect on the *P*(*t*), and the *θ_p_* parameter has a significant effect on the *P*(*t*), which can accelerate the negative emotion healing effect.

### 4.7. Analysis of the Influence of Risk Degree Coefficient a

**Figure 17:**
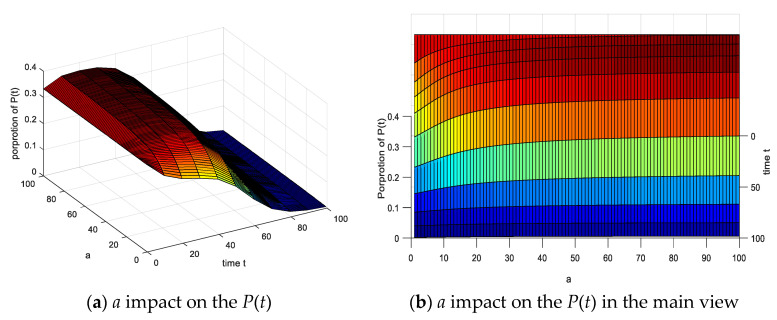
Risk coefficient *a* impact on the *P*(*t*).

The parameters of this section are set as follows. The proportion of various emotional subjects is the same as above. As can be seen from Figure 17: (1) When *a* from 0 to 100, the *P*(*t*) gradually rises, and the corresponding peak value increases at the same time. (2) The proportion of the rising level of the negative emotion group changes significantly in the range of *a* (0–20) with the increase of the risk degree coefficient *a*. (3) The proportion of the rising proportion in the range of 20–100 is not significant. It is indicated that as the coefficient of panic *a* increases, *P*(*t*) in the group have intensified, the existence of *P*(*t*) inhibits negative emotional infections when the risk degree coefficient reaches 20, and the rejection is prominent. The results are consistent in the results set in the paper.

## 5. Conclusions

Because of the occurrence of emergencies, the emotional changes among individuals tend to affect the development of events. It is very important to adjust the mood of the people to maintain social stability in emergency. This paper constructs the emotional infection model (U-SOSPa-SPSOa). Through numerical simulation, the following conclusions are drawn:

(a) Adding an emotionally stable group *U*(*t*) with control effect can inhibit the pessimists. By regulating the efficiency of emotionally stable groups with control, it can effectively control the proportion of pessimists in the group after the occurrence of an emergency, and thus avoid large-scale group turmoil or riots.
(b) The initial negative emotions below 0.34 belong to medium and low risks, and *P*(*t*) increase steadily, while above 0.34 belong to high-risk states. In this state, the speed of external rescue should be accelerated to fully regulate *P*(*t*) infections in time.
(c) In the three kinds of infection process, the influence of contact infection process is less than that of spontaneous infection and spontaneous recovery process. The spontaneous infection process can promote the emotional infection obviously, which shows that the spontaneous infection of group greatly affects the emotional infection process of group. Therefore, in reality, we should pay more attention to the spontaneous infection process of emergency events, strengthen effective safety publicity and training, improve the group’s drill skills in responding to public events, strengthen the intervention role of emotionally stable groups *U*(*t*), communicate information and confidence in a timely manner, and focus on the emergency skills of group when making emergency policies in order to control and improve the response.
(d) *θ_o_* has the least influence on the *P*(*t*), and its change range is small. At the same time, under the action of *θ_p_*, the *P*(*t*) reaches the stable state first. Controlling *θ_p_*, can effectively suppress infection of negative emotional groups.
(e) Increased risk factor *a* can promote pessimists, but below the critical level, it will increase the infection rate for pessimists, and slow down the infection rate above the critical value.

In addition, there are still some shortcomings in this research. One possible limitation of this study is the incompleteness of the social network dataset used. Because the Framingham Heart Study was not designed as a study of social networks, no attempt was made to capture all of a person’s important social contacts. However, even if under-sampling of real-world contacts did occur in the FHS Network, it does not change our results qualitatively. Future research should focus on the technology of integrating descriptive models within the optimization framework. For example, the probability of infection under different conditions and different events needs further analysis and research. Further research is needed in order to improve the environmental characteristics of specific situations and establish a mechanism of emotional infection of immune groups in combination with specific scenarios. The study of empirical cases is still an important work in the future.
